# Decylubiquinone Inhibits Colorectal Cancer Growth Through Upregulating Sirtuin2

**DOI:** 10.3389/fphar.2021.804265

**Published:** 2022-02-01

**Authors:** Jinlian Li, Shuting Zheng, Ting Cheng, Yuanyuan Li, Xiaobin Mai, Guangchun Jiang, Yongxia Yang, Qianqian Zhang, Jiangchao Li, Lingyun Zheng, Lijing Wang, Cuiling Qi

**Affiliations:** ^1^ Institute of Basic Medical Sciences, School of Life Sciences and Biopharmaceutics, Guangdong Pharmaceutical University, Guangzhou, China; ^2^ Guangdong Province Key Laboratory for Biotechnology Drug Candidates, School of Life Sciences and Biopharmaceutics, Guangdong Pharmaceutical University, Guangzhou, China

**Keywords:** decylubiquinone, colorectal cancer, sirtuin 2(SIRT2), tumor growth, tumor metastasis

## Abstract

Colorectal cancer (CRC) is one of the leading causes of cancer-related death worldwide. Decylubiquinone (DUb), a coenzyme Q10 analog, was reported to inhibit breast cancer growth and metastasis by us. However, the influence of DUb on CRC remains unclear. Herein, we found that DUb significantly inhibited CRC growth in the patient-derived xenograft (PDX) and CT26 xenograft models. DUb was further identified to significantly suppress CRC cell proliferation, colony formation, migration and invasion in a dose-dependent manner, while not inhibiting CRC cell apoptosis from flow cytometry assay. Sirtuin2 (SIRT2), a member of the sirtuin protein family, plays a critical role in growth and metastasis in various cancers. Moreover, DUb inhibited CRC progression by upregulating SIRT2. These findings reveal that DUb has the potential to a novel drug for the treatment of CRC by inhibiting CRC cell proliferation.

## Introduction

Colorectal cancer (CRC) is the third most common cancer worldwide and the second leading cause of cancer death (Azizi et al., 2020). Globally, approximately 1.8 million cases were diagnosed in 2018 (Azizi et al., 2020). In the United States, it is estimated that nearly 150,000 new cases are diagnosed as CRC and 52,000 people die each year (O'Leary et al., 2020). The 5-year survival rate is 90% for CRC diagnosed at an early stage compared with 13% for those diagnosed at a late stage (Sébert et al., 2019). Furthermore, despite consistent improvements in screening strategies and the development of more effective treatments, the 5-year overall survival rate for CRC remains poor (Danese and Montagnana, 2017). In addition, CRC cells often develop resistance to chemotherapeutic drugs, resulting in relapse and poor patient prognosis (Liu et al., 2019). As such, the development of innovative drugs for CRC is urgently required (Brody, 2015).

The ubiquinone Q_10_ analog decylubiquinone (DUb) with a 10-carbon side chain and a methyl group at the end can travel into mitochondrial membranes and inhibit activation of the mitochondrial permeability transition (MPT) (Lenaz et al., 1997; Armstrong, et al., 2003). It has been reported that DUb has a potent inhibitory effect on the prevention and treatment of diseases linked to oxidative stress (Murad et al., 2007). Emerging evidence has indicated that prolonged mitochondrial permeability transition pore (MPTP) opening causes mitochondrial energetic dysfunction and apoptotic and necrotic cell death (Kwong and Molkentin, 2015; Bonora et al., 2016). DUb was also found to induce PTP-dependent cell death in Clone-9 and MH1C1 cells (Devun et al., 2010). A report uncovered that the combination treatment of EDL-360 with DUb effectively suppressed glioma tumorigenesis by inducing cell death (Hosni-Ahmed et al., 2014). In addition, DUb inhibits breast cancer cell growth and metastasis by regulating the ROS/p53/BAI1 pathway (Cao et al., 2020). These findings suggested that DUb played an important role in the progression of some cancers and is associated with tumor-induced angiogenesis. The effect of DUb on CRC growth and metastasis has not yet been investigated.

Sirtuin 2 (SIRT2), a NAD^+^-dependent protein deacetylase (Feldman, et al., 2012), is mainly located in the nucleus, cytoplasm, and mitochondria (North et al., 2003; Kitada et al., 2019). SIRT2 was found to be a critical regulator of a variety of cancer processes including tumor growth, invasion and metastasis (Wang et al., 2019). It has been reported that SIRT2 expression is downregulated in ovarian cancer (Sun et al., 2019), prostate cancer (Damodaran et al., 2017), non-small cell lung cancer (Xu et al., 2015) and colorectal cancer (F. Du et al., 2020). Meanwhile, accumulating studies have indicated that elevated expression of SIRT2 was also associated with the progression of gastric cancer (Li et al., 2018), hepatocellular carcinoma, and basal-like breast cancer (Zhou et al., 2016). In addition, a study showed that high SIRT2 expression leads to a better prognosis for elderly patients with CRC (Lee et al., 2020) due to its antitumor activity and it acting as a potential therapeutic target for CRC (Du et al., 2020).

In the present study, we reported that DUb inhibited CRC growth by upregulating SIRT2. These findings demonstrated that DUb may be a promising therapeutic drug to inhibit the CRC growth by upregulating SIRT2.

## Materials and Methods

### Cell Lines and Cell Culture

The human CRC cell line (LoVo and HCT116) and mouse colon cancer cell line (CT26) were purchased from the Shanghai Institute of Cell Biology of the Chinese Academy of Sciences (Shanghai, China). These cells were grown in RPMI-1640 medium (Gibco, Carlsbad, CA, United States) containing 10% FBS (Gibco, Carlsbad, CA, United States) and 1% penicillin/streptomycin and incubated at 37°C in a humidified atmosphere with 5% CO_2_.

### MTT Assay

Cell viability following DUb treatment was determined using the MTT assay. In brief, CRC cells (1.0×10^3^ cells/well) were seeded in 96-well plates and treated with different concentrations of DUb, and control cells were treated with DMSO. After 96 h of DUb treatment, 10 µl of 5 mg⁄mL MTT was added to each well for an additional 4 h of incubation at 37°C. The medium was subsequently removed, and 100 µl of DMSO was added to the cells. Plates were agitated gently for 10 min at 37°C, and the absorbance at 570 nm was measured using a microplate reader (Molecular Devices, West Berkshire, United Kingdom). Half maximal inhibitory concentration (IC50) values were calculated from the inhibitory curves.

### Colony Formation Assay

For the colony formation assay, cells were seeded into 6-well plates with the quantity of 800 cells per well and coated with the indicated concentrations of DUb or DMSO the next day. Finally, the cells were allowed to grow for another 7 days, fixed with 4% paraformaldehyde, and stained with 1% crystal violet. The colonies (defined as any colony with ≥50 cells) were manually counted.

### Analysis of Apoptosis by Flow Cytometry

The apoptotic cells were detected with an Annexin V-FITC/PI apoptosis detection kit (Beyotime, Shanghai, China). Briefly, HCT116 and LoVo cells (1.0×10^3^ cells/well) were added into 6-well plates and treated with various concentrations of DUb for 96 h. Next, the cells were suspended in 200 μL of Annexin V binding buffer and incubated with 5 μL of FITC Annexin V and 10 μL of PI solution for 30 min in the dark. The percentage of apoptotic cells was analyzed by flow cytometry.

### Cell Migration and Invasion Assay

Transwell chambers with an 8-μm pore size were used to analyze cell migration without Matrigel coating, and chambers were coated with Matrigel (BD Biosciences San Jose, CA, United States) to analyze cell invasion in a 24-well plate. Cells (1.0 × 10^5^ cells/well) were dispensed into the upper chamber with 200 μL of serum-free medium containing DUb or DMSO, and the lower compartment was filled with 600 μL of medium containing 10% FBS. After 16 h (for cell migration assay) or 18 h (for cell invasion assay) of incubation, migrated or invaded cells on the lower layer of the membrane were fixed with 4% paraformaldehyde and stained with 1% crystal violet. The number of migratory or invasive cells was counted from six randomly selected 20-fold fields. Each experiment was performed three times.

### Tumor Models and DUb Treatment

Fresh specimens (2 mm^3^) of human CRC were orthotopically transplanted into 6-week-old athymic nude mice via axillary incisions. Athymic nude mice were intraperitoneally treated with DUb (5 mg/kg, Sigma, St Louis, MO, United States three times per week for 3 weeks) or DMSO for 54 days. CT26 CRC cell line was injected subcutaneously into the flanks of 6-8-week-old BALB/c mice. CT26 xenograft mice were treated intraperitoneally with DUb (5 mg/kg, Sigma) or DMSO every day since the tumor could be observed with the naked eye for 12 days. The body weights of mice were weighed during DUb or DMSO treatment. The length and width of tumors were measured every 2 days with a caliper, and the tumor volumes were calculated following the formula: length × width^2^ × 0.52. After 54 days or 12 days, the tumors were isolated for weighing and histological analysis.

### Immunohistochemical Staining

The tumor specimens were fixed with 4% paraformaldehyde, embedded in paraffin, and sectioned at 3 μm. After de-parraffinized in a xylene-ethanol series, the sections were blocked with 3% H_2_O_2_, and incubated with anti-Ki67 (Abcam, London, United Kingdom) or anti-SIRT2 (BOSTER, Wuhan, Hubei, China) antibodies overnight at 4°C. The next day, HRP-conjugated secondary antibodies were added to the sections and stained with DAB. The nucleus was counterstained with hematoxylin. For Ki67 quantitation, the number of Ki67 positive cells was counted in a 400 × field and showed as a percentage of the total cells per field. The proliferation index means the percentage of proliferating cells. SIRT2 expression was quantified using an image analysis program Image Pro-Plus 6.0 (IPP, version 6.0, Media Cybernetics).

### Quantitative Real-Time PCR

Total RNA was isolated with TRIzol reagent (Invitrogen, Carlsbad, CA, United States) from cells treated with DUb (45.84 µM or 28.11 µM) or DMSO, then reverse transcribed into cDNA according to the manufacturer’s protocol. Subsequently, reverse-transcription of RNA by PCR was performed using a Takara SYBR real-time PCR kit for the target gene. GADPH was used as an internal control, and the relative expression level was determined using the 2^−ΔΔCt^ analysis method. The specific primers were as followed: SIRT2, (For): 5′-TTC​AAG​CCA​ACC​ATC​TGT​CA-3′, (Rev): 5′-TCC​ACC​AAG​TCC​TCC​TGT​TC- 3’; GAPDH, (For): 5′-GGAGAA ACC TGCCAAGTATG-3′, (Rev): 5′-TTACTCCT TGGAGGCCATGTAG-3’.

### Western Blotting

HCT116 and LoVo cells were treated with different concentrations of DUb. The total proteins were extracted and lysed with RIPA lysis buffer (Thermo Scientific, Scotts Valley, CA, United States), subjected to 10% SDS PAGE (20 μg/lane) and transferred to PVDF membranes (Millipore, Billerica, MA, United States). Then, the membranes were blocked with 5% nonfat milk and incubated with rabbit anti-mouse or anti-human SIRT2 polyclonal antibody (BOSTER, Wuhan, China) and anti-GAPDH (eBioscience, CA, United States) at 4°C overnight. After the incubation with HRP conjugated secondary antibody, the PVDF membranes were subsequently subjected to immunoblotting analysis using the ECL immunoblotting kit (Beyotime Institute of Biotechnology, China) according to the manufacturer’s protocol. The relative protein expression levels were analyzed using Image-ProPlus 6.0 software (Media Cybernetics, Inc., Rockville, MD, United States).

### Silencing SIRT2

The siRNA of SIRT2 or control siRNA were designed and purchased from RiboBio Inc. (Guangzhou, Guangdong, China). LoVo cells were seeded into 6-well plates and treated with DUb. After 96 h, the cells were transfected with siRNA using Lipofectamine 3000 (Invitrogen, Thermo Fisher Scientific) by following the manufacturer’s protocol. The cells were subsequently prepared for use in further experiments.

### Statistical Analysis

All statistical analyses were performed with the GraphPad Prism 5 software package (GraphPad Software, CA). For two group comparisons, the results were compared using a two-tailed Student’s t-test when the prerequisites (independence and normal distribution) were satisfied. For multiple group comparisons, the results were compared using a one-way ANOVA followed by Bonferroni’s post-hoc test. A *p* value <0.05 or <0.01 was considered significant or very significant. Data are expressed as the mean ± standard error for each group.

## Results

### DUb Inhibits CT26 Xenograft Tumor Growth

**FIGURE 1:**
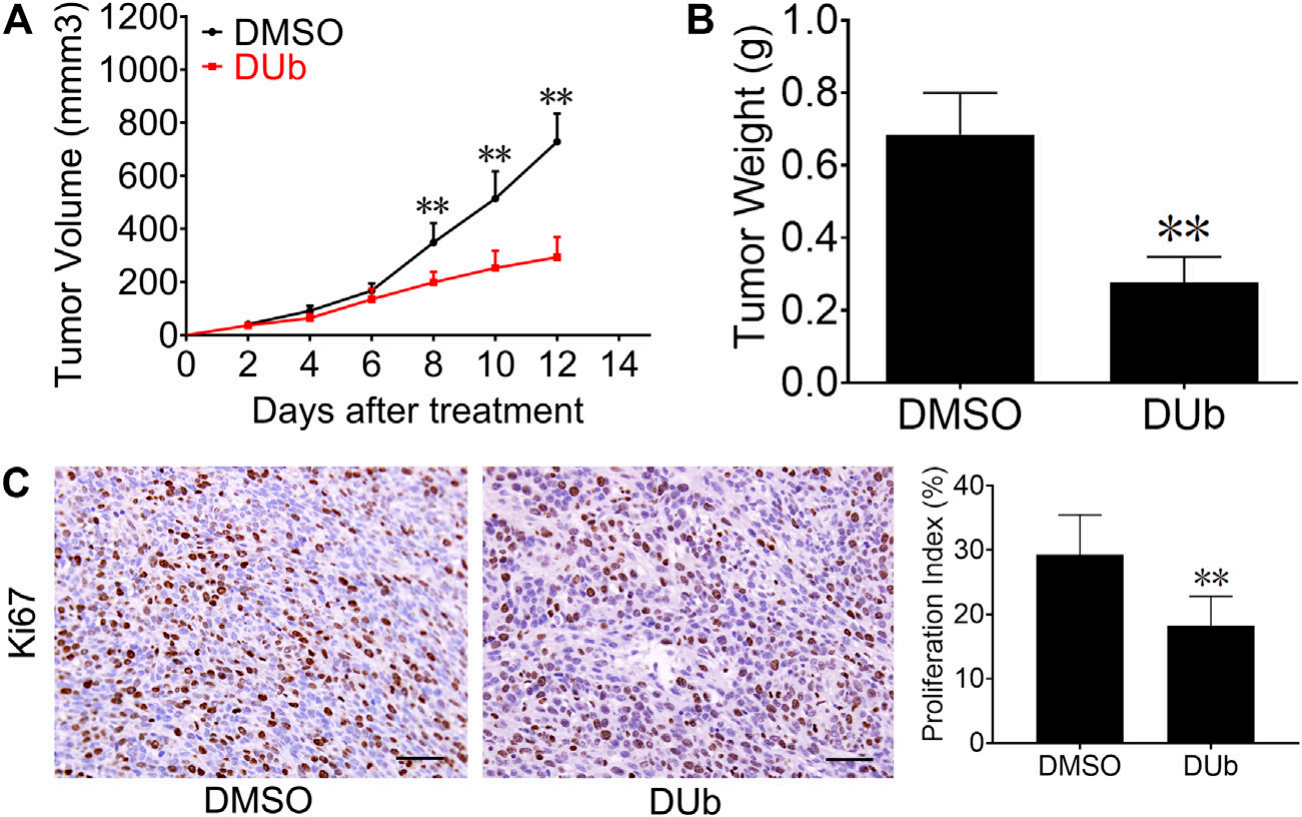
DUb inhibits CRC growth in CT26 xenograft tumor models. **(A)** The volume of subcutaneously transplanted tumors in the DUb treatment group (*n* = 9) was smaller than that in the control group (*n* = 7). **(B)** The tumor weight treated with DUb (*n* = 9) was lighter than that of the control group (*n* = 7). **(C)** The immunohistochemical image of Ki67-positive cells in the tumor tissues showed that the proliferative ability in tumor tissues treated with DUb (*n* = 7) was decreased. Scale bar = 50 µm in C. **p < 0.01*.*

To investigate the effect of DUb on CRC growth, we employed a mouse xenograft model with mouse CRC CT26 cells. DUb (5 mg/kg) or DMSO was injected every day for 12 days after subcutaneous inoculation of CT26 cells into the flanks. DUb was found to significantly attenuate CRC growth (Figure 1A) and weight (Figure 1B). In addition, DUb significantly decreased cell proliferation in tumor tissues as detected by immunohistochemical staining for Ki67 (Figure 1C), which can be used as an index of cell proliferation. Furthermore, there was no significant difference in body weights of mice between DUb group and DMSO group (Supplementary Figure S1A). Importantly, no obvious abnormal changes in histological structure of important organs were caused by the administration of DUb or DMSO for 12 days (Supplementary Figure S1C). These data suggest that DUb inhibits CRC growth by impeding tumor cell proliferation.

### DUb Inhibits Human-Derived Xenograft Tumor Growth

**FIGURE 2:**
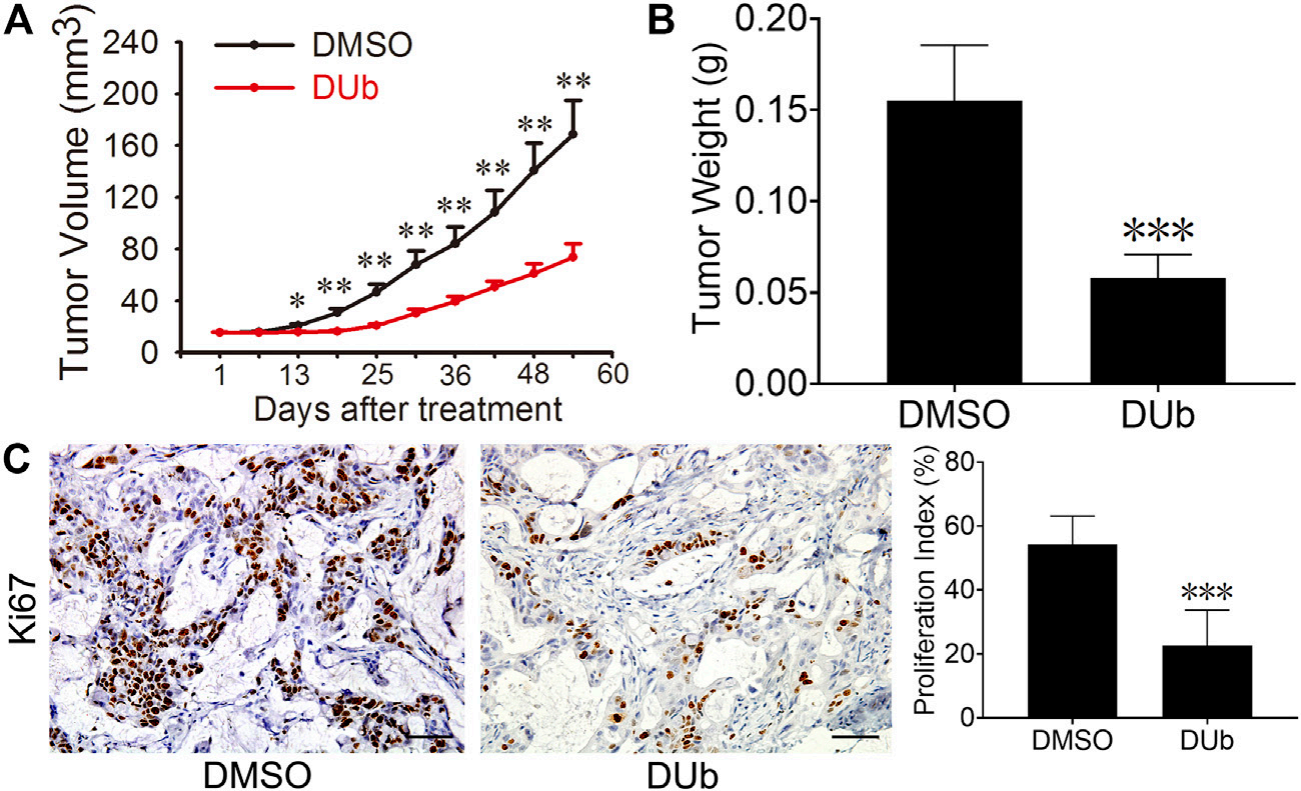
DUb inhibits the CRC growth in the patient-derived xenograft (PDX) tumor models. **(A)** The tumor volume in the DUb treatment group (*n* = 6) was smaller than that of the control group (*n* = 6). **(B)** The tumor weight in the DUb treatment group (*n* = 10) was lighter than that of the control group (*n* = 11). **(C)** The immunohistochemical image of Ki67-positive cells in the tumor tissues showed that the proliferation ability of the tumor tissues treated with DUb (*n* = 3) was decreased. Scale bar = 50 µm in C. *p < 0.05; **p < 0.01; ***p < 0.001*.*

Given that DUb suppresses mouse-derived CRC growth, we speculated that DUb can also inhibit human-derived CRC growth by attenuating tumor cell proliferation. To this end, we established a xenograft CRC model in 6-week-old athymic nude mice using fresh specimens of human CRC. Compared with DMSO group, DUb significantly decreased tumor volume (Figure 2A) of human-derived xenograft tumor. After nude mice were sacrificed 54 days after DUb treatment, the tumors were isolated and weighed. Compared to DMSO group, DUb treatment group demonstrated that tumor weight in DUb-treated mice was significantly reduced (Figure 2B). The results of IHC staining against Ki67 antibodies indicated that cell proliferation index in DUb-treated xenograft tumor was significantly reduced as compared with the control (Figure 2C). Furthermore, there was no significant difference in body weights of mice between DUb group and DMSO group (Supplementary Figure S1B). These results show that DUb reduces CRC growth by suppressing tumor cell proliferation *in vivo*.

### DUb Suppresses CRC Cell Proliferation and Colony Formation

**FIGURE 3:**
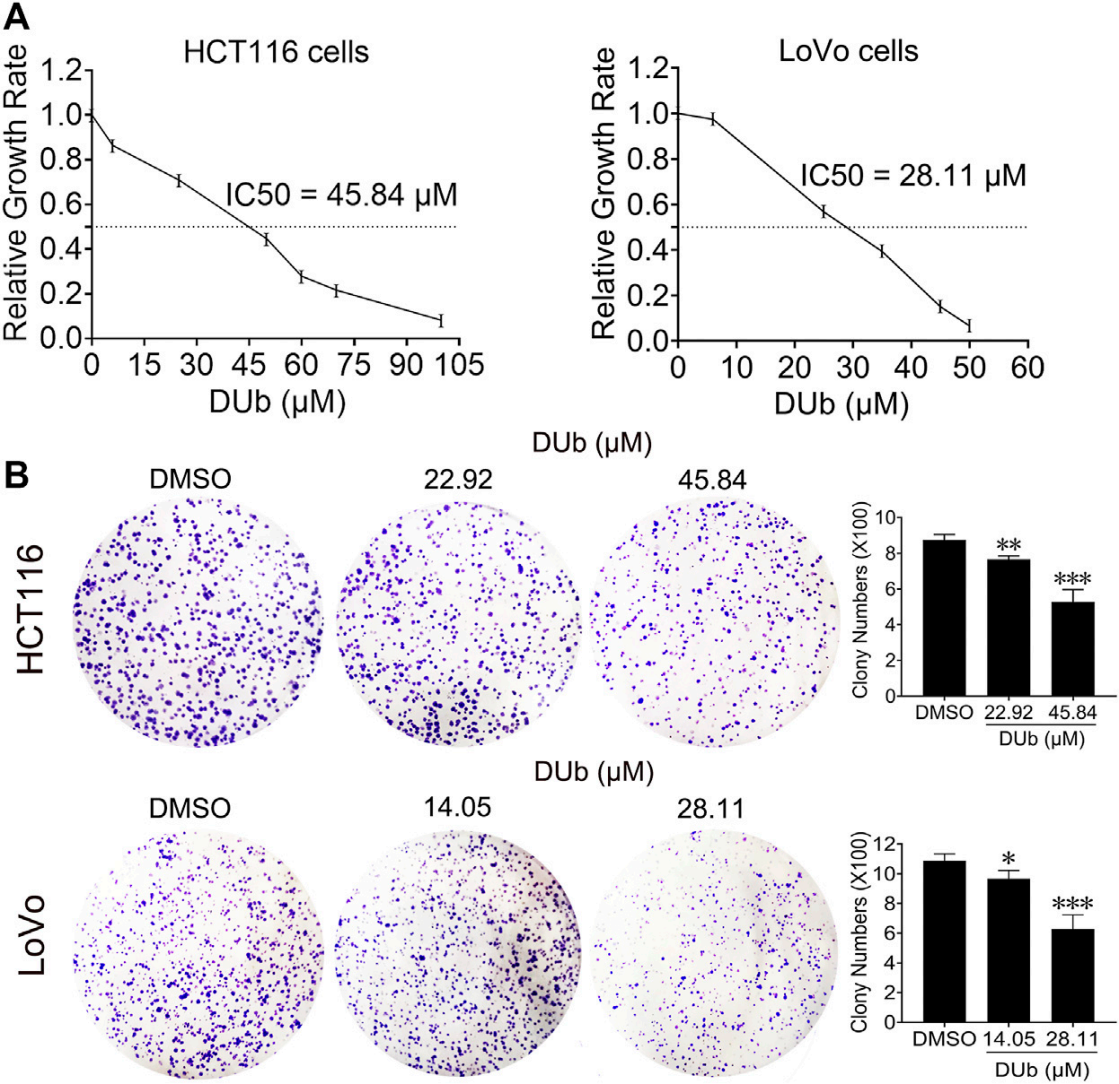
The effects of DUb on proliferation and colony formation of CRC cells. **(A)** After HCT116 and LoVo cells were treated with DUb or DMSO for 96 h, DUb suppressed the proliferation of HCT116 and LoVo cells (*n* = 12). **(B)** HCT116 and LoVo cells were treated with different concentrations of DUb (*n* = 4) and colony formation assay was performed. The colonies were stained with crystal violet solution and captured. DUb-treated HCT116 and LoVo cells displayed fewer colobies compared with DMSO group (*n* = 4). *p < 0.05; **p < 0.01; ***p < 0.001.

To explore the effect of DUb on CRC cell proliferation, HCT116 and LoVo cells were treated with different concentrations of DUb for 96 h, and the cell viability was detected by the MTT assay. We demonstrated that DUb significantly reduced the proliferation of HCT116 and LoVo cells in a dose-dependent manner (Figure 3A). The IC50 values of DUb were calculated to be 45.84 µM for HCT116 cells and 28.11 µM for LoVo cells (Figure 3A). Therefore, 22.92 and 45.84 µM DUb treatments for HCT116 cells and 14.05 and 28.11 µM DUb treatments for LoVo cells were used for the subsequent studies. DUb, a PTP inhibitor, has a key effect on cell apoptosis. To explore whether DUb affects CRC cell apoptosis, the CRC cells were stained with of annexin V-FITC/PI and detected by flow cytometry after DUb or DMSO treatment for 96 h. There is no difference in the percentage of apoptotic cells between DUb and DMSO group (Supplementary Figure S2). To further confirm the effect of DUb on colony formation of CRC cells, the colony formation assay was performed using HCT116 and LoVo cells. We found that HCT116 and LoVo cells treated with DUb displayed fewer colonies as compared with the control (Figure 3B). These data shows that DUb significantly inhibits CRC cell proliferation and colony formation.

### DUb Suppresses CRC Cell Migration and Invasion

**FIGURE 4:**
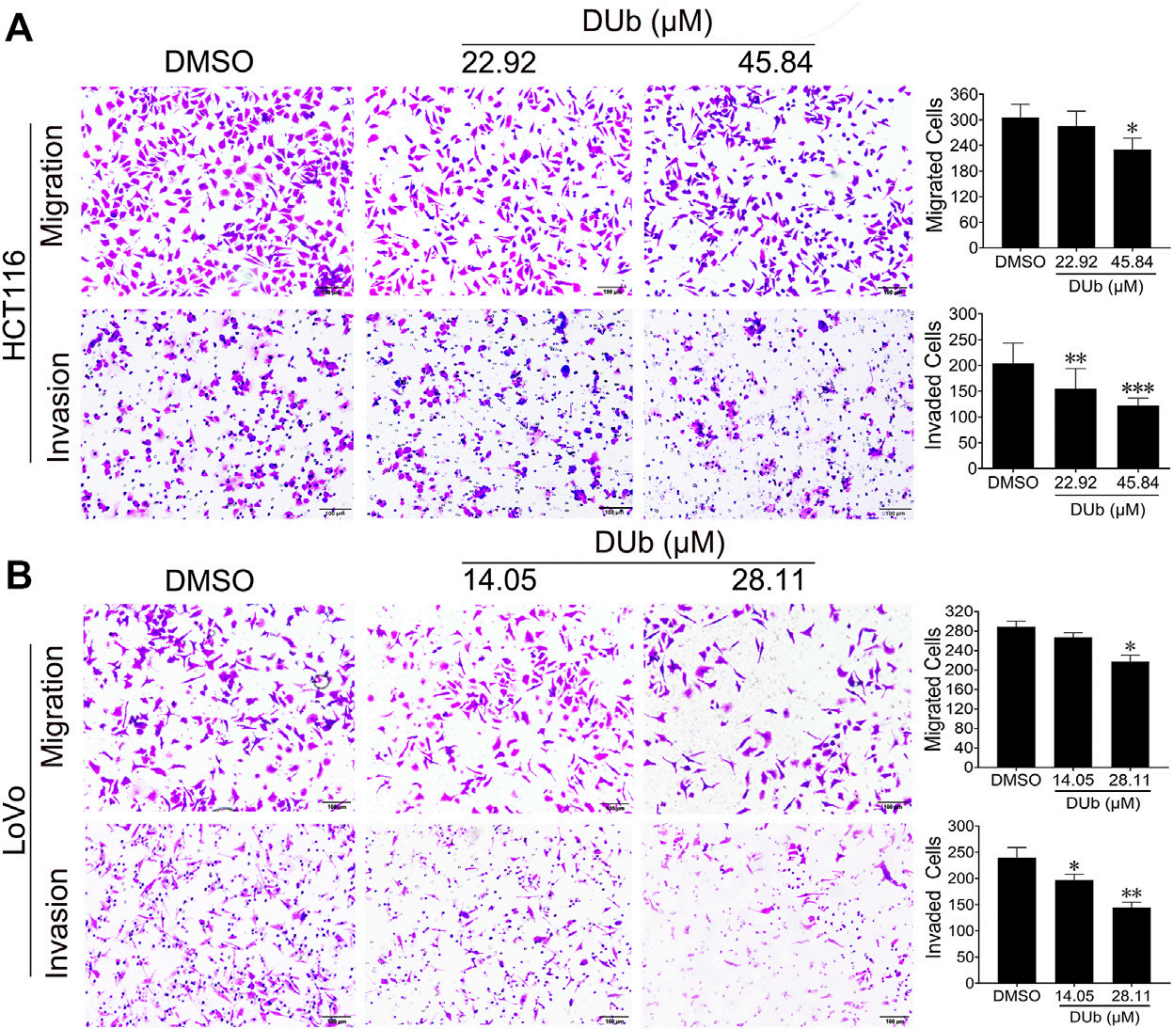
The effects of DUb on migration and invasion of HCT116 and LoVo cells. **(A)** DUb (*n* = 9) significantly inhibited the migration and invasion of LoVo cells. **(B)** DUb (*n* = 9) significantly inhibited the migration and invasion of LoVo cells. Data are presented for at least three independent experiments. Scale bar = 100 µm in **(A,B)**. *p < 0.05; **p < 0.01; ***p < 0.001*.*

Given that DUb attenuated CRC growth through inhibiting tumor cell proliferation, we explored whether DUb inhibits CRC cell migration and invasion. We found that DUb significantly inhibited the CRC cell migration and invasion inside Boyden Chambers in a dose-dependent manner (Figure 4). These results shows that DUb reduces the migratory and invasive abilities of CRC cell.

### DUb Upregulates SIRT2 Expression

**FIGURE 5:**
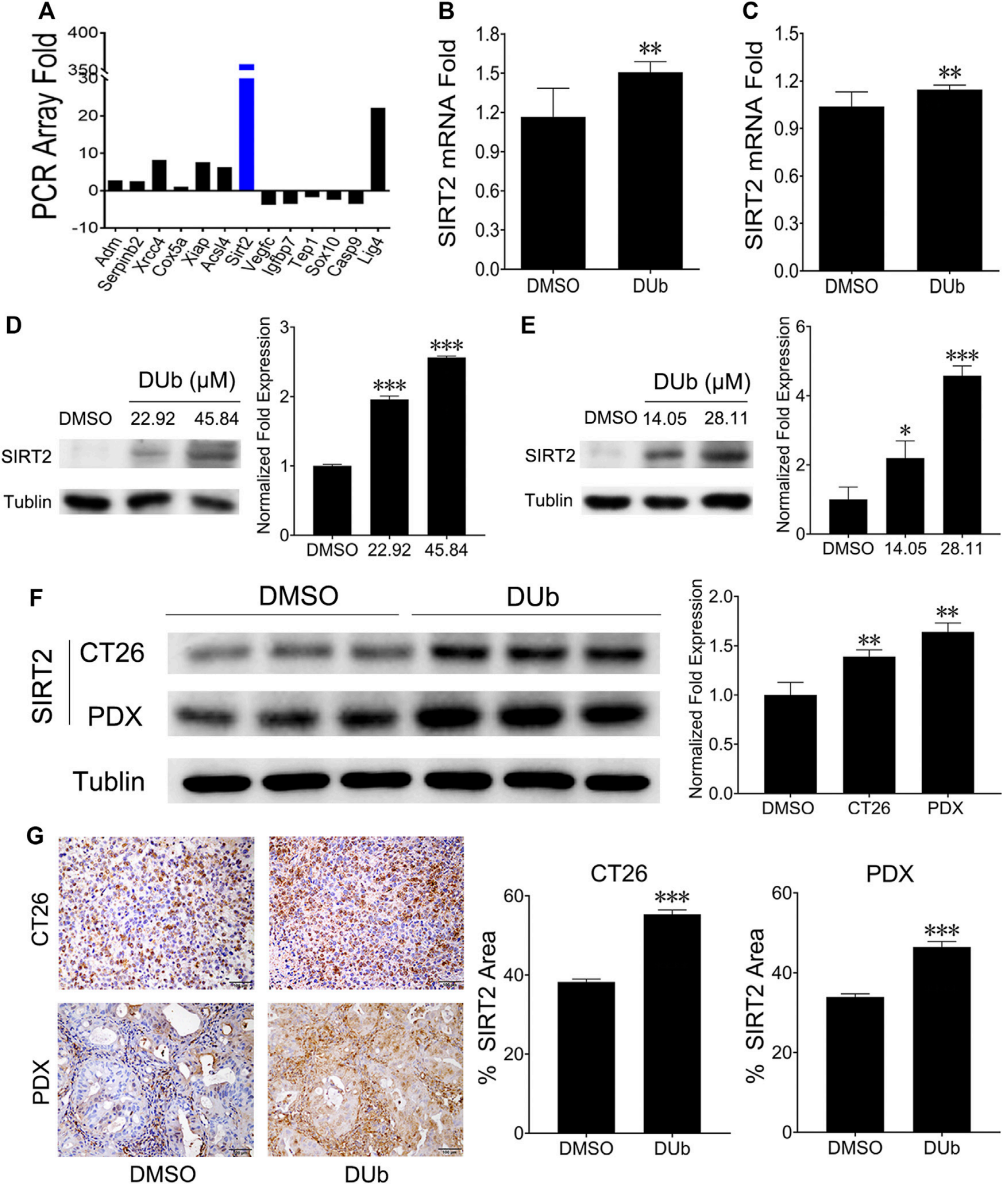
DUb increases the expression of SIRT2. **(A)** qRT-PCR array analysis showed that SIRT2 expression was upregulated in CRC cells treated with DUb. **(B,C)** qRT-PCR was further investigated to determine the qRT-PCR array results (*n* = 8). DUb significantly induced mRNA expression of SIRT2 in HCT116 **(B)** and LoVo cells **(C)**. **(D)** DUb markedly induced the protein expression of SIRT2 in HCT116 cells. **(E)** DUb markedly induced the protein expression of SIRT2 in LoVo cells. **(F)** The results of western blotting showed that DUb markedly increased the protein expression of SIRT2 in the CT26 xenograft tumors and patient-derived xenograft (PDX) tumors (*n* = 3). **(G)** The results of immunohistochemical staining showed that DUb markedly increased the protein expression of SIRT2 in the CT26 xenograft tumors and patient-derived xenograft (PDX) tumors (*n* = 3). Scale bar = 100 µm in **(G)**. *p < 0.05; **p < 0.01; ***p < 0.001.

To determine the mechanism of DUb inhibiting CRC growth, a qRT-PCR array was performed. SIRT2 expression was found to be markedly upregulated (Figure 5A). qRT-PCR was further investigated to determine whether SIRT2 expression was increased in DUb-treated HCT116 and LoVo cells (Figures 5B,C). To further explore the role of DUb in the protein expression of SIRT2, western blotting was performed. Western blotting results showed that SIRT2 expression was significantly upregulated in DUb-treated HCT116 and LoVo cells (Figures 5D,E) and CRC tissues treated with DUb (Figure 5F). Furthermore, the results of IHC staining against SIRT2 antibodies shows that SIRT2 expression in the DUb-treated xenograft tumor is significantly increased as compared with control.

### DUb Inhibits CRC Growth by Upregulating SIRT2

**FIGURE 6:**
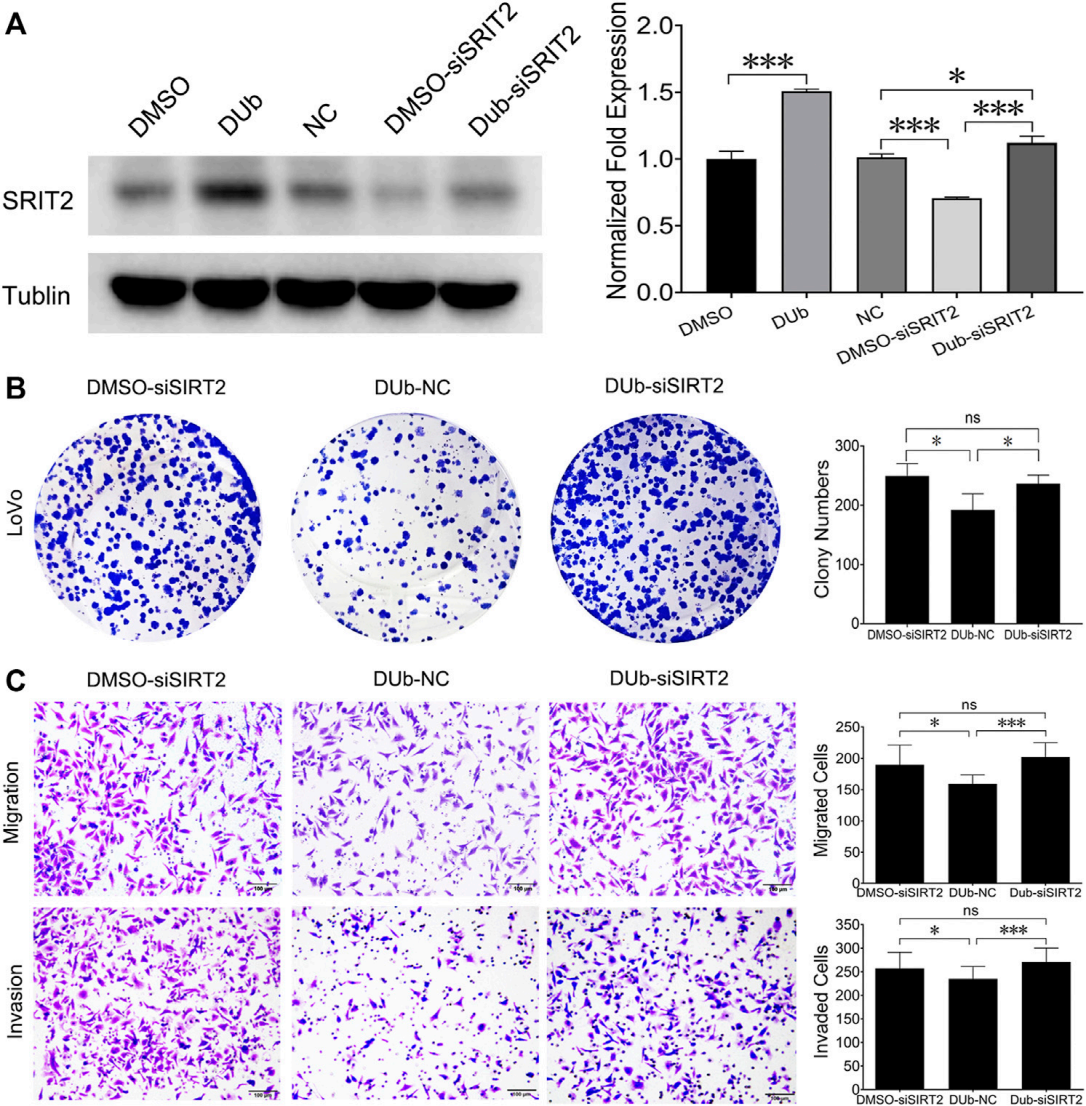
DUb inhibits the colony formation, migration and invasion of LoVo cells throuth upregulating SIRT2. **(A)** DUb did not change SIRT2 expression in SIRT2-silenced LoVo cells (*n* = 3). **(B)** DUb did not affect the ability of LoVo cells to form colonies after SIRT2 was silenced (*n* = 4). **(C)** DUb did not affect the ability of LoVo cells to migrate (*n* = 6) and invade (*n* = 6) after SIRT2 was silenced. Data are presented for at least three independent experiments. Scale bar = 100 µm in C. *p < 0.05; ***p < 0.001.

Given that DUb upregulated SIRT2 expression, we speculated that DUb inhibited CRC growth by upregulating SIRT2. We firstly found that DUb upregulated the SIRT2 expression in LoVo cells, but DUb did not affect SIRT2 expression after SIRT2 was silenced (Figure 6A). We investigated the significance of SIRT2 in CRC growth by detecting whether silencing SIRT2 can promote LoVo cell colony formation, migration and invasion. These results showed that silencing SIRT2 promoted LoVo cell colony formation, migration and invasion. LoVo cell colony formation, migration and invasion were rarely affected by DUb after SIRT2 was silenced (Figures 6B,C).

## Discussion

In this study, we report an important finding that DUb, a coenzyme Q10 analog, can potently suppress CRC cell proliferation, migration and invasion by up-regulating SIRT2 expression. Therefore, DUb attenuates CRC growth in xenograft mouse models with CRC CT26 cells and human CRC tissues.

CRC is one of the most diagnosed cancers with a rapidly increasing trend (Tomasello et al., 2017). Despite the high incidence of colorectal cancer worldwide, very few effective drugs are available in clinic. Recent study demonstrated that DUb combined with either EDL-360 or thialysine significantly inhibited the growth of human acute leukemia or glioma (Hosni-Ahmed et al., 2014). We have previously reported that DUb inhibited breast cancer growth and metastasis using spontaneous breast carcinoma MMTV-PyMT mice and xenograft models with breast carcinoma 4T1 cells and MDA-MB-231 cells (Cao et al., 2020). However, the effect of DUb in CRC growth has never been investigated. Our results show that DUb markedly suppresses CRC growth in xenograft mouse models with colorectal carcinoma CT26 cells and human-derived xenograft tumor. Furthermore, our studies have demonstrated that the inhibitory effect on CRC growth induced by DUb treatment was attributed to attenuate tumor cell proliferation instead of tumor cell apoptosis. Our findings provided direct evidence that DUb has the potential to be an effective therapeutic agent for CRC.

To explore how DUb inhibits CRC cell proliferation, qRT-PCR analysis was performed to determine cancer-related genes using DUb-treated CRC cells. We found that DUb inhibits CRC cell proliferation through up-regulating SIRT2 in CRC cells. It has been reported that SIRT2 plays critical roles in non-small cell lung cancer growth and metastasis through inducing p53 acetylation and reducing the transcriptional activity of p53 (Wang et al., 2019). Furthermore, SIRT2 has an important role in breast cancer, hepatocellular carcinoma and other tumors (Chen et al., 2013; Jing et al., 2016; Zhang et al., 2020). SIRT2 overexpression was also found to significantly suppress CRC cell proliferation, invasion and migration (Du et al., 2020). In addition, SIRT2 was associated with poor prognosis of CRC (Hu et al., 2018). These findings clearly demonstrated that SIRT2 is involved in various tumor progression including CRC, and SIRT2 might be a novel prognostic biomarker for CRC. We also found that DUb upregulated SIRT2 expression in DUb-treated HCT116 and LoVo cells and CRC tissues treated with DUb. It was further demonstrated that DUb inhibited LoVo cell proliferation, migration and invasion by upregulating SIRT2. Thus, the accumulating evidence showed that DUb may target the SIRT2 in CRC cell.

DUb as the coenzyme Q10 analog may induce mitochondrial dysfunction, thereby affecting tumor metabolism. Therefore, DUb may inhibit CRC growth by affecting the metabolism of colorectal cancer.

Taken together, as an important finding of the current study, DUb is determined to suppress CRC growth in xenograft mouse models with CRC CT26 cells and human CRC tissues. Its therapeutic efficacy is dominantly related to its ability of inhibiting tumor cell proliferation rather than CRC cell apoptosis. While, SIRT2 is regarded as one of the dominant targets for the anti-tumor cell proliferative action of DUb. Our results in this study demonstrates that DUb may be the potential agent for the treatment of CRC through upregulating SIRT2.

## Data Availability

The original contributions presented in the study are included in the article/Supplementary Material, further inquiries can be directed to the corresponding authors.
