# Supplementary material for: Decylubiquinone Inhibits Colorectal Cancer Growth Through Upregulating Sirtuin2
Source: Front Pharmacol. 2022 Feb 1;12:804265. doi: 10.3389/fphar.2021.804265 (PMC8844026; doi:10.3389/fphar.2021.804265)

**Supporting Information**

## Decylubiquinone Inhibits Colorectal Cancer Growth Through Upregulating Sirtuin2

***Jinlian Li^1,ϯ^, Shuting Zheng^1,ϯ^, Ting Cheng^1^, Yuanyuan Li^1^, Xiaobin Mai^1^, Guangchun Jiang^1^, Yongxia Yang^1^, Qianqian Zhang^1^, Jiangchao Li^1^, Lingyun Zheng^1^, Lijing Wang^1^,*, Cuiling Qi^1, 2^****

*^1^Institute of Basic Medical Sciences, School of Life Sciences and Biopharmaceutics, Guangdong Pharmaceutical University, Guangzhou, Guangdong 510006, China; ^2^Guangdong Province Key Laboratory for Biotechnology Drug Candidates, School of Life Sciences and Biopharmaceutics, Guangdong Pharmaceutical University, Guang Zhou, Guangdong 510006, China*

***Correspondence:**

Lijing Wang

wanglijing62@163.com

Cuiling Qi

qicuiling12345@163.com

Jinlian Li ^ϯ^ and Shuting Zheng ^ϯ^

^ϯ^ These authors have contributed equally to this work and share first authorship.

**Table of Contents: Supplemental Figures**

**Figure S1.** The effects of DUb on the body weight of mice and histological structures of main organs. Page 2

**Figure S2.** DUb did not affect the colorectal cancer cell apoptosis. Page 3

**Figure S1.** The effects of DUb on the body weight of mice and histological structures of main organs. The mouse xenograft models with CT26 cells and human-derived CRC tumor were established, and then were treated with DUb or DMSO. The body weights of mice were weighed during DUb or DMSO treatment. DUb did not affect the body weight of mice both in the CT26 xenograft tumor **(Supplemental Figure S1A)** and human-derived xenograft tumor **(Supplemental Figure S1B)**. Importantly, no obvious abnormal changes in histological structure of important organs were caused by the administration of DUb or DMSO in the CT26 xenograft tumor model **(Supplemental Figure S1C)**.

**

**

**Figure S2.** DUb did not affect the colorectal cancer cell apoptosis. Colorectal cancer cells were treated with DUb or DMSO for 48 h. After apoptotic cells were stained with annexin V-FITC/PI, flow cytometry was used to analyze and quantify the apoptotic cells.


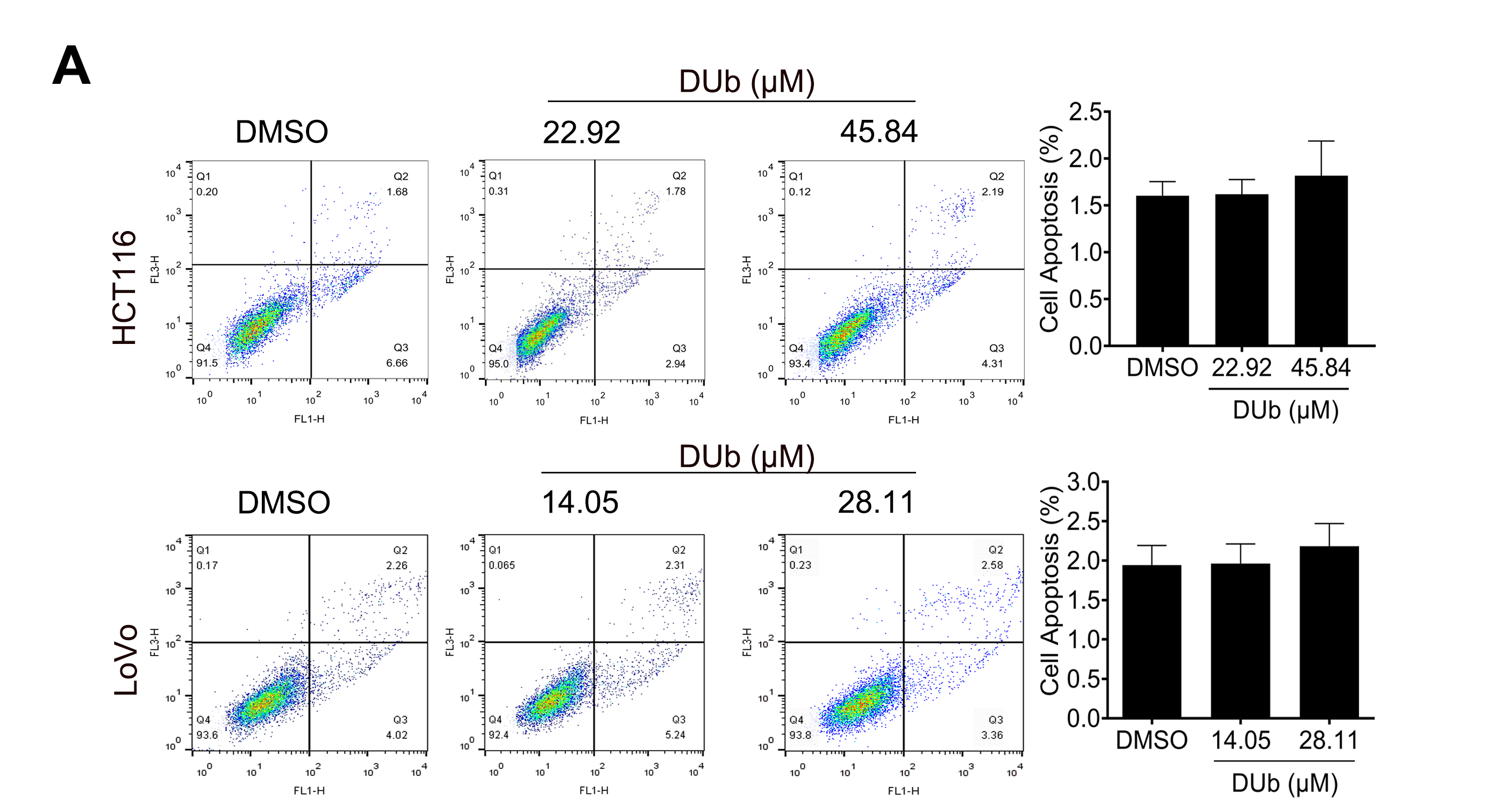

Supplement: Supplementary file 3 [file DataSheet1.doc]
